# The paradox of necessary uncertainty: Psychopathy, welfare and Munchausen Syndrome in 1950s England

**DOI:** 10.1017/S026988972500047X

**Published:** 2025-06-23

**Authors:** Chris Millard

**Affiliations:** History of Medicine and Medical Humanities, School of History, Philosophy and Digital Humanities, University of Sheffield

**Keywords:** Psychopathy, Munchausen Syndrome, welfare state, personality disorder, sickness benefit

## Abstract

The cluster of psychiatric concepts that includes “personality disorders,” “psychopathy” and “moral insanity” has long been controversial and uncertain. This article investigates the concept of “psychopathy” in 1950s England and shows how this ambiguity is not a flaw or failure in the concept but absolutely necessary for the role it carries out: policing broad areas of social life. A case of Munchausen syndrome (a type of “psychopathy”) in the late 1950s still functions as a precedent in the welfare system today, denying claimants sickness benefit, “closing a loophole,” and exemplifying the usefulness of this uncertainty.

Taxonomies normally try to avoid ambiguity and uncertainty. If categorizations contain ambiguous or ambivalent criteria, this is lamented, if sometimes acknowledged as inevitable. In one cluster of categories: personality disorders, antisociality and—in particular—psychopathy, I will argue that ambiguity is *not only a central characteristic but a fundamental necessity*. I will use the case of William Spencer Davies, a lorry driver in 1950s England diagnosed with Munchausen syndrome (thought to be a form of psychopathy), to disqualify him from sickness benefits. (This case, as an important precedent, remains a functioning part of the British welfare state.) Through this case, I will show how uncertainty performs this necessary role. Psychopathy (and Munchausen syndrome) police the borderlands between medical pathology and legal criminality, in the amorphous space that is now called “antisocial behavior.” This involves focusing on how psychopathy functions, more than how it is defined. As we shall see, nobody is very comfortable defining it, but it has clear and obvious material effects.

Not very much is known about William Spencer Davies. Despite this, there is an enormous paper trail concerning his actions in the records of the UK Ministry of Pensions (now subsumed into the Department of Work and Pensions (DWP)). Davies submitted many claims for sickness benefit during the 1950s, claiming that he was unable to work due to investigations and tests carried out in NHS hospitals. The paper trail does not show him breaking any law due to this behavior, but reflects the fact that he was considered to be highly deceptive and untruthful. In this context, he was the subject of two tribunal decisions that still guide welfare policy to this day. These decisions have been made public (known as “Reported Decisions”), and are included in bound volumes for the guidance of Welfare Commissioners.

William Spencer Davies is paradoxical: obscure, but with a huge bureaucratic footprint. His case sheds light upon the function of *ambiguity* in the categories of “personality disorders,” “antisocial behavior” and “psychopathy.” The interactions between psychiatry, the courts, claims on healthcare and welfare resources, and diagnoses of personality disorder continue to generate controversy.^1^ The issues that generate anxiety around Davies have not gone away.

Recent academic analysis of the categories of “psychopathy,” “antisociality” and “personality disorder” is frank about the conceptual difficulties that surround them. Historian Greg Eghigihan calls psychopathy a “drifting concept,” and sociologist Martyn Pickersgill characterizes ideas of antisociality as “ontological anarchy” (see Eghigihan 2015, 283; Pickersgill 2014, 143). This present article has a simple argument: the uncertainty, drift, anarchy, and confusion that surrounds these definitions is not the result of incompetence, flawed reasoning or incomplete knowledge. Instead, psychopathy *needs* to be uncertain from a medical or legal perspective in order to perform one of its basic functions: the policing of so-called antisocial behavior that does not otherwise break the law or contravene state regulation. Psychopathy, antisociality and personality disorder are all historically linked, managing a grey area between—and beyond—mental illness, criminality and morality.

This article focuses upon a distinctive time period within this prolonged effort to manage behavior that defies easy categorization. During the 1950s, there were extensive discussions in Britain of the concept of “psychopathy,” which explicitly addressed the difficulties around certain “antisocial” behaviors from legal and medical perspectives. New understandings of mental health and mental illness were circulating alongside a new and vastly extended system of social and welfare provision. These discussions show how the concept of “psychopathy” only *seems* incoherent if it is viewed from a narrowly legal or medical perspective. Viewed more broadly, “psychopathy” functions as an explanation for persistent behavior that is largely not illegal, and only uncertainly pathological. The category thus helps to *police broad areas of social life* and social institutions. It is only when viewed narrowly that it seems confused.

Counterintuitively, forcing this concept to be narrow and precise leads to ambiguity and imprecision. Viewing it as a broad catch-all helps to bring it into focus. It is only when we accept the medical or legal claims made in its name—when we assume that it is indeed a label of pathology, or of criminality, and expect it to function like a standard psychiatric diagnosis or legal definition—that it appears incoherent.

Psychopathy at this point in history is a “social diagnosis,” and this much is clear from existing work on the issue (e.g. Jones 2015, 188). Again, this does not mean it is necessarily incoherent or logically flawed: the concept only seems incoherent if one is judging the category by orthodox medical or legal criteria. In order to fulfil this “social role,” psychopathy must *continually operate beyond* the standard diagnostic and legal parameters of the time, creating the impression of incoherence. Seen from this new angle, psychopathy is still an exceptionally problematic category, but has a sort of flexible coherence. We need a historically-informed sense of its function (i.e., what does this label *do* and how does it do it?) if we are to subject it to meaningful criticism.

To clarify the abstraction of “policing broad areas of social life” the article will, having laid out these contexts, pivot from theories about psychopathy to the specific example of William Spencer Davies. His diagnosis of Munchausen syndrome—a rare set of behaviors described as a variant of psychopathic personality—shows how this policing works in practice.

The term Munchausen syndrome was coined in 1951, and referred to the persistent faking of illness by individuals presenting at Accident and Emergency (A&E) departments of hospitals. The syndrome was named by London hematologist and polymath Richard Asher in *The Lancet* in 1951. In his article, he describes people who travel widely and present deceptive or self-induced symptoms at many different hospitals. He claims that “most doctors have seen” the condition, but little has been written about it, describing three varieties: acute abdominal symptoms, the hemorrhagic or bleeding type, and the neurological type (with fainting, headaches or fits) (Asher 1951). Broadly, in published correspondence over a number of years, Munchausen patients became differentiated (inconsistently and uncertainly) from hysterics and malingerers: the former because their physical symptoms are not consciously simulated and the latter because they fake illness with a specific aim in view. In contrast, Munchausen patients were seen to be conscious and deliberately deceptive, but initially without any clear aim or motive.^2^

Asher’s paper expresses his concern at these dramatic patients, who stagger into A&E holding their stomachs, groaning in pain, fainting, or bleeding from mouth or ears, only to be discovered as fakers either hours or sometimes days later. Some are recognized by hospital porters, others become aggressive and assault hospital staff, and some tell improbable stories and get found out under close scrutiny. They almost always leave (often after an indignant emotional outburst) rather than accept psychiatric help. Some turn up at another hospital days later, sometimes at the other end of the country.

Asher’s article sparked an extended correspondence (in both *The Lancet* and the *British Medical Journal*) from doctors seeking to protect NHS funds by publicizing the patients they have encountered. Some went as far as to propose a central blacklist of such patients (Irvine 1955). Asher’s examples were even picked up by *Time* magazine in the United States (Anon 1951).

Though both male and female cases are reported, and two of Asher’s initial three examples are women, my sense of the published medical literature is that the majority of Munchausen cases reported in the Anglophone medical literature during this period are male. Whilst deceptiveness, manipulation and emotional outbursts might well fit a feminized stereotype (and certainly predominate in the related, later category of Borderline Personality Disorder), these cases in the 1950s are strongly coded as vagrants (another proposed name for this condition is “hospital hoboes” (Clarke and Melnick 1958)) or con-artists. They are also stereotypically described as simmering with resentment and barely-controlled aggression. The most celebrated, repeated and colorful cases (e.g. Leo Lamphere (“The Indiana Cyclone”), or Samuel McIlroy) are men.^3^ However, multiple examples of other reported illness-faking behavior have been swept up under different frames of reference, without the Munchausen term (which emphasizes the dramatic and charismatic tall tales). Medical commentary on fictitious fever (pyrexia), induced bleeding from anticoagulant use, or what is called “polysurgical addiction” during this period is much more feminized or at least less male-dominated.

William Spencer Davies was diagnosed with Munchausen syndrome in the late 1950s, in order to “protect” social welfare funds (i.e. deny them to a particular kind of claimant). This involved the intersection of medical expertise and a welfare tribunal. The diagnosis thus policed social provision (welfare) and provided an exemplar for the social diagnosis of psychopathy in general in this period. To be clear: the conduct of this one particular person led to sickness benefit rulings that *still inform welfare policy to this day* (although under the updated rubric of “personality disorder”).^4^

## The nature of psychopathy’s definitional chaos

There is a vast literature on uncertainty in medicine, which seeks to reduce, embrace, or otherwise manage uncertainty in medical practice (see Mather, Millard and Sabroe 2022). The academic study of medical uncertainty can usefully be traced back to Renee Fox’s work (1957). However, most of that work either seeks to analyze uncertainty in order to reduce it (because it is seen as an impediment to good healthcare) or to have clinicians manage it more effectively (because uncertainty is inevitable). Sally Mather’s study of uncertainty in medical practice found vanishingly few positive associations with uncertainty, but some have described uncertainty as helpful in eventually getting to the correct prognosis, leaving space for hope, or being thrilling and stimulating for the clinician involved.^5^ None of these are close to the analysis of uncertainty here, which focuses upon uncertainty’s necessity, functioning to exclude patients from various kinds of support simultaneously.

As might be expected, is almost impossible to define “psychopathy” in a few short lines. Personality disorders are described by the latest *Diagnostic and Statistical Manual of Mental Disorders* of the American Psychiatric Association as an “enduring pattern of inner experience and behavior the deviates markedly from the norms and expectations of the individual’s culture, is pervasive and inflexible, has an onset in adolescence or early adulthood, is stable over time, and leads to distress or impairment” (APA, 2022). Other definitions are rather more moralizing and threatening. Forensic psychiatrist Hugues Hervé describes “instrumentally impulsive individuals with poor behavioral controls who callously and remorselessly bleed others for purely selfish reasons via manipulation, intimidation, and violence … [with an] ability to easily supersede morality for personal gain and do so without remorse” (Herve’ 2017, 25).

During the 1950 and 1960s in Britain psychopathy was most often understood to refer to persistent “antisocial” conduct *not* accompanied by defects of intelligence (what was at the time called “subnormality” or “feeblemindedness”). The conduct is also not thought attributable to any other mental disorder (Fox 1964, 194). David Kennedy Henderson’s classic definition argues that “psychopathic states” describe people “who throughout their lives, or from a comparatively early age, have exhibited disorders of conduct of an anti-social or asocial nature, usually of a recurrent or episodic nature” (Henderson 1939, 16–17).

Almost all commentary on the category of “the psychopath” mentions its incoherence or how it escapes definition. David Jones notes “the absence of a solid definition” (Jones 2015, 236), and Susanna Shapland observes that when “psychopath” began to be used in a recognizably modern sense in Britain after the Second World War, it “still had so much uncertainty and ambiguity clinging to it” (Shapland 2018, 98). Likewise, Pickersgill argues that antisocial personality disorder has a history of “contestation and uncertainty” (2009, 670).

The differences between “personality disorder,” “psychopathy” and “Munchausen syndrome” are complicated enough in the present, but historically, the shifting of meaning under various terms makes this even more chaotic. Broadly (and very crudely), psychopathy is sometimes seen as an outdated term for personality disorder,^6^ but sometimes also a specific (and extreme) sub-type of personality disorder. The specific meaning of “psychopathic” as it is used today is not even one hundred years old, as Janet Weston has pointed out:

The term “psychopathic” did not carry the same meaning in the early twentieth century as it later acquired. Until the 1930s, it commonly referred in general terms to the borderland between sanity and psychosis, or a generic illness of the mind. Gradually, it came to signify a specific variety of mental disorder, and acquired a noun—the psychopath. (Weston 2019, 65)

Pickersgill has noted how even in current guidelines (where “psychopathy” is still used) ideas of Antisocial Personality Disorder (ASPD) and psychopathy are sometimes treated as synonymous and sometimes distinct (in the *same* guidelines!). Pickersgill subjects these to a careful close reading, showing how in some cases “psychopathy” is an important but small subset of ASPD, with different treatment recommendations, and at other times it is treated at synonymous with it. Further, these “ambiguities are now such an entrenched part of everyday psychiatric discourse that they have become invisible” (Pickersgill 2009, 669–70). Adding to the chaos, Munchausen syndrome itself is now an outdated term, having mostly been replaced by the term “factitious or induced illness” (FII), but it remains associated with a diagnosis of personality disorder (NHS Website [n.d.]). During the period addressed in this paper, Munchausen was reliably and indeed repetitively explained as being a form of psychopathy.

## Uncertainty as necessary rather than simply inevitable

It is useful to expand upon one of Pickersgill’s arguments around “ontological anarchy” in order to clarify what is being argued here with regard to “necessary uncertainty.” When analyzing the search for biological characteristics that correspond to psychopathy or antisociality, he argues that:

I characterize the multiplicity of psychiatric praxis that has sought to define the mark of antisociality as a form of “ontological anarchy.” I regard this as an *essential feature* of the search for biological and other markers of an unstable referent, positing that uncertainties endure—in part—precisely because of attempts to build consensus regarding the ontology of antisociality through biomedical means. (Pickersgill 2014, 143, emphasis added)

So, an anarchic sense of what antisociality *is* (its ontology) is an “essential feature.” This does sound close to what I want to argue about “necessary uncertainty,” but there is a key difference. Something being essentially uncertain means that the uncertainty is irresolvable, and I am entirely in agreement on this point.^7^ However my argument is much less concerned with ontology than with function. My argument is that psychopathy and antisociality *need* to be uncertain in order to *do what they do* as categories.

This overlaps with uncertainty being their essence, but not completely. Pickersgill’s argument appears entirely agnostic on the question of whether this category’s uncertainty is necessary to its functioning. He argues that the category *is* essentially anarchic and I am arguing that it *needs* to be that way. Uncertainty is a feature, not a bug, of psychopathy and personality disorder. In Pickersgill’s reading, the uncertainty is a problem to be solved, even if that solution is implied (in places) to be impossible. In my reading (which is not in direct conflict with Pickersgill’s) the uncertainty is necessary.

Pickersgill also flags dangers of reification—and of effacing or eclipsing the uncertainty in psychopathy and personality disorder. He argues, quite rightly, that “the ontological uncertainty so characteristic of personality disorder in the mid-20th century has endured” (Pickersgill 2009, 669). A quarter of a century ago, German Berrios lamented that it “remains unclear to the clinician which of the obscurities besetting the concept of personality disorder are man-made and which are inherent to it” (1993, 14). Even further back, Henry Werlinder’s landmark study called the diagnosis of psychopathy “the object of disagreement and debate of seldom-witnessed intensity,” citing a researcher from the 1950s who called this term a “chamber of horrors of obsolete theories and a swamp of conceptual confusion” (1978, 9).

During the 1950s, this sentiment was repeatedly expressed by almost all who pronounce upon the topic. Aubrey Lewis, the most influential psychiatrist of his day, argued that “every textbook of psychiatry discusses this abnormality, but almost always ambiguously because the authors do not make clear why it should be regarded as an illness” (1953, 119). The category would become absolutely central to the deliberations and evidence given to the Royal Commission on the Law Relating to Mental Illness and Mental Deficiency (Percy Commission) which convened between 1952 and 1957.

The Royal Medico-Psychological Association (RMPA; forerunner of the Royal College of Psychiatrists) gave evidence to the Commission that psychopaths are “difficult to classify,” speaking further of “difficulties in medical diagnosis” (Royal Commission 1954a, 287–88). The Magistrates’ Association lamented that “an attempt to clarify the relationship between mental defectiveness and psychopathic personality is apt to reveal only the confusion of thought that exists even among psychiatrists” (Royal Commission 1954b, 363; see also the British Medical Association evidence on this point: Royal Commission 1955a, 1054–5).

From both a psychiatric and legal point of view, there is no coherence, no agreement. The diagnosis cannot fit securely into either context. As we will see below, it operates *between them* not inside either. In fact, when questioned, the BMA witness went further: “your psychopath is *ex hypothesi* [according to the hypothesis] a person who is not susceptible to medical diagnosis. … I have never seen a general definition of a psychopath by behaviour, when the behaviour was not also observable in someone who was not a psychopath” (ibid. 1095). Thus, according to the *entire hypothesis* of what constitutes a psychopath, medical diagnosis does not work. Except of course, it absolutely “does work” in the sense of having powerful effects on those diagnosed with it. This is precisely the kind of ambiguity and paradox that will be expanded upon below when discussing Munchausen syndrome: where a *lack* of any mental disorder to explain behavior is paradoxically a flag for a particular kind of mental (personality) disorder.

Barbara Wootton, celebrated sociologist, criminologist and magistrate, makes much of the negotiations around the new Mental Health legislation in her classic Social Science and Social Pathology (1959). She glosses Aubrey Lewis’ assessments from the early 1950s, claiming that “the volume of the literature on the subject of psychopaths is rivalled only by the depth of the confusion in which this literature is steeped” (Wootton 1959, 250). Wootton is chiefly concerned with notions of (legal) responsibility, and her forensic analysis sums up the state of “psychopathy”:

The psychopath makes nonsense of every attempt to distinguish the sick from the healthy delinquent by the presence of or absence of a psychiatric syndrome, or by symptoms of mental disorder which are independent of his objectionable behaviour. In his case no such symptoms can be diagnosed because it is just the absence of them which causes him to be classified as psychopathic. … He is in fact, *par excellence*, and without shame or qualification, the model of the circular process by which mental abnormality is inferred from anti-social behaviour while anti-social behaviour is explained by abnormality. (ibid)

The whole concept seems circular and paradoxical. However, the Percy Commission Report (1957) states: “We are convinced from the evidence we have received that is it not difficult for doctors to recognise these characteristics and to make a diagnosis that is readily acceptable to an intelligent layman. … The difficulty of describing them [psychopaths] is a difficulty of language rather than diagnosis” (quoted in Walker and McCabe 1973, 218). In fact, as criminologists Nigel Walker and Sarah McCabe argued in 1973, “the Commission were putting a bold face on their inability to agree any specific definition” (ibid.).

The issue of defining psychopathy arose repeatedly in the 1957 parliamentary debate on the report. To pick just two examples, Dr Edith Summerskill, Labour MP for Warrington, and member of the Socialist Medical Association, asked, “who can be certain of recognising the dividing line between extreme eccentricity and some pathological deviation?,” adding that “we now propose officially to recognise a category of mental disorders [psychopathy] which, hitherto, has tended to baffle authority”.^8^ Summerskill’s mention of eccentricity points suggestively to an idea of social transgression—the idea that people who persistently refuse to abide by social convention are in some cases indistinguishable from those labelled psychopaths. Kenneth Robinson, Labour MP for St Pancras North, first Chair of the National Association of Mental Health (NAMH, later MIND), and Minister of Health 1964–1968, lamented that “it is a pity that the Royal Commission was unable to agree on a definition. Having been unable to agree on a definition of a psychopath, it was an even greater pity that they made a virtue of their failure and said that no definition should be included in the law.”^9^ As Shapland has argued, “the Percy Commission … felt compelled to put psychopathy at the heart of its discussions, and recommended the introduction of the term into the statute book for the first time.” This was extremely unusual, and not only because in a move aptly described as “astonishing,” the Percy Commission “recommended the term be enshrined in law, but not defined” (Shapland 2019, 106–7). In the end this was not accepted by Parliament, and the following definition was inserted into the Mental Health Act 1959: “In this Act ‘psychopathic disorder’ means a persistent disorder or disability of mind … which results in abnormally aggressive or seriously irresponsible conduct on the part of the patient, and requires or is susceptible to medical treatment.”^10^

The evidence given to the Percy Commission had convinced them that psychopaths were an urgent social problem to be addressed, partly because they were, in Shapland’s terms “at the frontline of what was socially acceptable, unwittingly testing the boundaries of social norms,” but also because “the psychopath” had become this menacing figure who inexplicably refused to conform to society’s norms, and took on much wider importance: “By defining the psychopath in societal terms, psychiatry had essentially distilled the ‘urgent’ problem of social upheaval into one public problem figure” (Shapland 2019, 103–4).

The definitional difficulty is painfully obvious. It is not that conflicting but internally coherent classifications leave us with a general picture that looks incoherent. The uncertainty and incoherence in the concept of psychopathy and personality disorder (this latter is a term used even in the 1950s and 1960s) is a central, baked-in part of the concept. It is clear that this uncertainty was well-recognized at the time: I am not saying anything new on that front.

Here it makes sense to refer to the preceding discussion around Pickersgill’s notion that the essence of psychopathy is “ontological anarchy.” Because nowhere in any of this commentary, either in scholarly historical or sociological work in the present or at the time, does anyone say that the uncertainty surrounding psychopathy is *necessary* for it to perform its function. Uncertainty might be seen as inescapable, but this is not the same as saying it is necessary. The closest we get in the primary documents is the BMA witness to the Percy Commission, who comments that the psychopath is *ex hypothesi*—outside of conventional medical diagnosis. In the secondary literature, the closest to our argument here is Shapland’s PhD thesis, which states: “The psychopath by definition had always sat in a ‘no man’s land’, both between sanity and insanity but also between medicine and law” (Shapland 2019, 106). Again, whilst Shapland is absolutely right, there is little argument here about the positive benefit or utility of this uncertainty, except to say: “psychopathy was too valuable a concept to relinquish. Its vagueness, non-specific aetiology and the considerable ground it was forced to occupy, all at various points considered weaknesses that meant the term should arguably have been abandoned, also allowed it to transcend fashion and disciplinary boundaries, and constantly reinvent itself to match the concerns of the day” (ibid. 109).

So again there is a sense of psychopathy being irresolvably positioned between medicine and criminal justice, as well as having extremely vague causes and treatments—but also a sense of being “valuable” because of this lack of resolution (or perhaps despite it). Weston has argued that:

When it comes to difficult psychiatric diagnoses such as psychopathy or Antisocial Personality Disorder, it may be that the disunity or “anarchy” within psychiatric thought, where many different perspectives coexist, is valuable in that it allows contested diagnoses to remain in use and to welcome ongoing research, even in the absence of any clear consensus as to exactly what they mean. (Weston 2019, 8)

Again, Weston cautiously (and plausibly) speculates on the “disunity” being valuable. Finally, Greg Eghigihan, using material from Germany, argues something similar related to the “success” of the term: that the “vagueness and plasticity of the diagnosis of psychopathy proved to be one of the keys to its success, as it was embraced and employed by social caseworkers, psychologists, welfare administrators, criminologists, policy makers, and the mass media” (Eghigihan 2015, 284).

Here then, is a clear sense of the value of disunity, vagueness, uncertainty or anarchy, because it allows the diagnoses to be used even when there is no agreement as to what they describe. This is still quite a “negative” benefit, in the sense that the uncertainty allows the diagnosis to continue to be used *despite flaws*. The uncertainty is still seen as produced by *defects* in the concept, but the uncertainty allows the category flourish in different contexts and in different ways.

This is not quite the emphasis that I want to promote here. I wish, instead, to focus on the value of the category’s uncertainty as neither medical, nor legal, but also both. On the one hand, it is able to disallow claims on welfare on the grounds of a (medical) syndrome or illness, which also do not allow the patient to avail themselves of mental healthcare or medical treatment, because this “illness” is in fact a character defect. On the legal side, as we have seen, as much as the British Medical Association believed it is “unwise to attempt” any definitional work because “such a definition might prove difficult to operate in a court of law” (Royal Commission 1955a, 1054–55) a definition was written into statute anyway, at the heart of the Mental Health Act, 1959. The question of who exactly benefits from this value-through-ambiguity is addressed further in conclusion, but it is enough to say here that this uncertainty can never be used by the patient, and is always deployed by those administering the healthcare, welfare, or criminal justice systems. Psychopathy works to disqualify a person on one ground (i.e. mental illness) without giving the protections of that category (by turning illness into a character disorder), even when no law has been broken.

The idea that the “psychopath” falls outside of conventional (medical, legal) categories, and is therefore neither straightforwardly pathological nor illegal begins to sound like Thomas J. Scheff’s argument around “residual rule-breaking.” Scheff argues in Being Mentally Ill: A Sociological Theory (1966) that when various behaviors transgress social norms, they are labelled as “ill-mannered, ignorant, sinful, criminal, or perhaps just harried, depending on the type of norm involved” (Scheff 1966, 55). Scheff goes on to say that if norm-violation falls outside of these categories, and especially if the transgressed norms are so universally held they are not normally articulated, “these violations are lumped together into a residual category: witchcraft, spirit possession, or, in our own society, mental illness” (ibid). However, even in this scheme psychopathy *again* evades description, because the psychopath is not securely mentally ill either, as we saw from Barbara Wootton’s discussion of psychopathy: “the psychopath makes nonsense of every attempt to distinguish the sick from the healthy delinquent by the presence of or absence of a psychiatric syndrome” (1959, 250).

One way to understand the positive value of this uncertainty and incoherence is by looking at another broadly agreed aspect of the concept in the 1950s and 1960s: the diagnosis is *social*. Jones relates that “it was a remarkably social understanding of the disorder that received a certain amount of official sanction, as manifest in the inclusion of ‘psychopathy’ in the 1959 Mental Health Act” (Jones 2015, 188). Pickersgill notes that psychopathy and personality disorders have been characterized as “disorders of sociality” (2014, 143). Thus the disorder is not—at this point—located in an individual’s biology, but more ambiguously in a set of behaviors and/or interpersonal relationships that imply a defective personality. Henderson, for instance, is clear about this “social” aspect in his 1939 book *Psychopathic States*. Then, in a 1942 article of the same name, he again claims that “those suffering from psychopathic states represent a group of struggling humanity, social misfits if you like” (Henderson 1942, 485).

Antisociality is central to the definition of psychopathy in this period. Janet Weston argues that Henderson and his close friend and collaborator Robert Dick Gillespie influentially conceptualized a person with “psychopathic personality” in the 1930s and 1940s “as one who was emotionally volatile, selfish, disinclined to shoulder responsibility, lacking judgement or foresight and often engaging in antisocial—although not necessarily criminal—behaviour” (Weston 2019, 65). Somewhat uncommonly in the early twentieth century, this psychopathic personality was “not unanimously thought to be the product of heredity” (ibid). Henderson and Gillespie’s conceptualization left much room for the potential impact of “faulty training and environment” on the genesis of this condition. However, even up to the end of the Second World War, psychopathic personality nevertheless “retained a strong affinity with inherited, constitutional or inborn traits” (ibid). This reading receded in the immediate postwar period in Britain.

Medicine in general, and psychiatry in particular, became much more interested in and oriented towards the social setting in the mid-twentieth century. The classic statement of this (from a highly Foucauldian standpoint) is David Armstrong’s *Political Anatomy of the Body* (1983), but everything—from general practice and emotional wellbeing to marriage guidance and attempted suicide—began to take environment, learning and social context into account (see, for example Oakley 1997; Hayward 2009; Chettiar 2012; Millard, 2015). In this context it is unsurprising that psychopathy’s hereditary aspect faded from the foreground.

Maxwell Jones, a highly influential psychiatrist and therapeutic community pioneer, described psychopathy in a memorandum to the Percy Commission as “social abnormality,”— a condition distinguishable from a “mental abnormality.” He also spoke, when questioned by the Commission, of a “concept of social defectiveness as … more realistic than any attempt at a diagnostic classification.” When questioned, he remarked that “[his] own feeling is that there is more hope of finding a definition related to social beahviour than to psychiatric classification” (Royal Commission, 1955b, 1230–31, 1235). The BMA opens its section on psychopathic states by talking of “mental abnormality,” which renders people “delinquent or otherwise anti-social” (Royal Commission 1955a, 1054), a formulation repeated by the RMPA (1954a, 287). The Magistrates’ Association also mentions that “a concept of ‘social inefficiency’ would help the definition” (Royal Commission 1954b, 363).

“Social” definitions are not inherently incoherent. Focus should be on the standards through which incoherence is judged. It is the strictly legal or medical standards that produce the incoherence. Indeed, those who discuss psychopathy in the 1950s seem relatively sure that outside of those bounds there is not really a problem of definition. Conservative MP Reginald Bennett takes refuge in an idea of common sense, noting that:

Two distinguished authors of a standard text book on psychiatry^11^ prefaced their book with this statement: I cannot define an elephant, but I know one when I see one. That I think is very apposite to trying to define a psychopath. Almost any psychiatrist—not just those who are very self-opinionated, but those working in all humility—will probably think that he will be prepared to diagnose a psychopathic personality when he meets it, but I doubt if he could define the condition to anybody else. (Hansard 1957, v.573 c.73).

So, there is a sense of coherence to the term, but it evades formal definition. Given that people also seem to agree that this concept has something to do with sociality, it follows that the coherence of this concept might be found in ideas of “the social setting,” rather than in abstract medical or legal definitions. “Psychopaths” become intelligible when they transgress social conventions or boundaries repeatedly.

Indeed, psychopathy in the 1950s was part of a much wider discussion—about the social setting, but also about policing social behavior. This ambiguity and its social consequences were discussed in a number of areas. A critical editorial in the medical magazine *Medical World* in 1958 argued that “the criteria of psychopathy are social not medical. Doctors should not be asked to act as the social conscience of society” (anon. 1959, 138). We can see this more explicitly in the Percy Commission evidence. One of the most revealing exchanges comes from Maxwell Jones’ evidence, given to the Commission in July 1955. The evidence begins with explicit acknowledgement of the link between antisociality and psychopathy: “I would like to put forward a plea for the treatment of the severe character disorder with antisocial trends. … I have in mind the sort of person who is frequently referred to as an aggressive or inadequate psychopath” (Royal Commission 1955b, 1229).

During the Percy commission hearings, Jones engaged in a back-and-forth exchange with one of the legal experts, (Richard) Meredith Jackson—a distinguished scholar who, in 1966, was made Downing Professor of the Laws of England at the University of Cambridge. It is in this discussion that the social policing aspect emerges most fully, as Jackson and Jones zoom out from concepts of treatment and certification and begin to discuss psychopathy in expansive social terms. Jackson, for instance, questioned Jones on the material he had submitted to the Commission: “It is implicit in … a good deal of your memorandum that you think the control which society exercises has to be recast from time to time” (ibid, 1237). Jones agrees with this, but Jackson then continues with a revealing example which is worth quoting at length:

If there is a certain kind of row [argument], it may mean that a person appears before the Court on some charge, a charge of assault, say, and then this machine works. Then there may be the kind of conduct that you refer to, lack of emotional control by adolescents and adults, the breaking up of family relationships, but because it does not happen to come within the particular category recognized by the law, it does not come before the Court. It seems to me what you are really after is something like a re-casting of certain kinds of prohibitions of the law to meet our present state of society. (ibid., 1238)

Jackson and Jones’ dialog casts the problem as one of transgressive behavior that falls outside the categories or institutions traditionally supposed to manage it. Jackson asks in this extract whether the law’s categories should be recast, and Jones responds emphatically in the affirmative: “that is an attractive way of looking at it”. Indeed, he goes further, adding: “I think you have seen very clearly my preference for social or sociological ways of looking at behaviour rather than by classification in the rigid framework of a doctor’s attempt at medical definition. I feel this is a much wider psychological problem than I feel competent to define”. Again, the behavior evades conventional categories of understanding. Jackson’s final, pithy response on the subject of psychopathy is: “So it is much more than a diagnosis?” to which Jones responds simply “Yes” (ibid., 1238).

Psychopathy entails zeroing in on behavior that is seen as explicitly falling through gaps in the various systems that police social relations and social behavior. This is exactly the *point* of psychopathy in the 1950s. What is clear throughout this exchange, between a scholar of jurisprudence and a psychiatrist, is that this category of “the psychopath” is broad, mapping onto social behavior that does not fall into the “rigid framework” of medical diagnosis, but also does not “come before the Court.” As we will soon see with the example of William Spencer Davies, psychopathy is also functionally useful when behavior falls between regulations of the welfare system.

In the final Percy Commission report, it is explicitly acknowledged that the sense of “psychopathy” has indeed been made broader: “We use the term ‘psychopathic personality’ in a wider sense than that in which it is often used at present and intend it to include any type of aggressive or inadequate personality which does not render the patient severely sub-normal … *but which is recognised medically* as a pathological condition” (Royal Commission 1957, 6 emphasis added). It is this medical recognition that we shall see is of central importance in the Davies case—making medical assessors aware that this is a legitimate diagnosis, but one that is *also illegitimate* for the purpose of claiming funds from the state, and is not really a mental illness either.

## Munchausen syndrome, psychopathy and Sickness Benefit

In the interests of specificity, instead of resting with the coherence of a broad term like “the social setting,” I want to focus on a particular variant of psychopathy (Munchausen syndrome) in a particular aspect of social provision—in this case, social welfare, particularly sickness benefit. Behavior that seems to fall outside restrictions to sickness benefit payments is policed and disciplined by an ambiguous but effective use of “psychopathy.” The ambiguity is absolutely central to its policing of social welfare resources. Before we get to those sickness benefit specifics, we need to recap some of the context to Munchausen syndrome, specifically around the presumed motives of those diagnosed with it, which help to understand its status (in the 1950s and 60s at least) as a variant of psychopathy.

Munchausen syndrome was not initially described as a variant of psychopathy, but became absorbed into that category as a way to understand the motives behind it. However, given the definitional uncertainty and confusion around anything to do with psychopathy, it would be more accurate to say that “psychopathy” helped shape the way Munchausen Syndrome was understood by absorbing and labelling, rather than resolving, the ignorance and incomprehension around Munchausen motivations.

Initially, Asher was unsure what causes people to behave in this way, providing a list of motivations he calls “scanty.” In his inaugural article, he hypothesizes that the behavior is rooted in a “strange twist of personality” or a vague “psychological kink” (Asher 1951, 339, 341). The key motivational sticking point for Asher is that these people are seemingly prepared to undergo multiple uncomfortable, painful even dangerous diagnostic tests, with the only discernible gain (so far as he can make out) being free board and lodgings (Asher 1951, 341). This motive was broadly considered—by Asher and by subsequent commentators—inadequate to account for the behavior.^12^

This lack of a motive can properly be called structural (rather than just something “unknown” about the condition) because it is the *lack* of discernible motive that differentiates Munchausen from malingering. In malingering, illness or injury is induced or simulated for a clear gain—typically the avoidance of military service or other work. One doctor summed up this differentiation in 1957. Retorting angrily to the blithe labelling of Munchausen patients as “malingerers,” he claimed that people “had better think again and a little more deeply about malingering. A person who pretends to be ill, and who obtains no objective gain by so pretending, is very ill indeed” (Bardon 1957, 1170). The other key factor in Munchausen is the *active* and presumably conscious procurement or fabrication of symptoms, which differentiates it from hysteria and hypochondria, where the patient is thought to believe their symptoms.

Initially, this uncertainty around motives was absorbed into psychopathy. There was one point of general agreement across the correspondence responding to Asher’s article: Munchausen patients are “psychopaths.” There is so much of this in the 1950s correspondence that three instances will suffice to demonstrate. Eric Frankel from Wanstead Hospital argued in 1951 that “these patients are invariably severe psychopaths, and their psychopathic personality requires treatment” (Frankel 1951, 911). Two doctors in 1958 expressed their relief that “these patients have at last been recognised as a special type of psychopathic personality” (Clarke & Melnick 1958, 6). Similarly, a doctor reporting from Charing Cross Hospital in 1960 mentioned an “underlying psychopathic constitution,” although the reasons for the development of Munchausen “remain uncertain” (Peyman 1960, 700–701). This, the correspondents concluded, is a particular case of psychopathy, with a particular kind of antisociality attached: the supposedly unfair exploitation of social welfare, in this case, the newly nationalized hospitals of the National Health Service.

The diagnosis of psychopathy and personality disorders—being in essence a diagnosis of *antisociality*—is related to the apparatus of the modern state and governance, insofar as the state attempts to police and regulate social environments and social relationships. David Jones argues that psychopathy and personality disorders “can be viewed as a creation of the modern state, most evidently constructed at the junction of the legal system and the welfare services” (2015, 235). Munchausen syndrome is fundamentally tangled up in the welfare state, and it emerges at one of the key entrance points to the National Health Service— Accident and Emergency (A&E) Departments. Thus, it is part of anxiety (from doctors) around the unfair exploitation of welfare resources, or public money.

Such anxiety-laced visions are not specific to this place or period, having been charted during the Great Depression of the interwar period (see Deacon 1976; Welshman 2006). However, A&E Departments are the most common and important place in the Munchausen discussions of the 1950s–60s. This is because A&E is, in this period, the only part of a hospital at which patients can legitimately present without a letter of referral from another doctor. This means that all initial assessments of illness are done by the relatively junior and overworked A&E staff.

## Disciplining William Spencer Davies with ambiguity

The case of William Spencer Davies does not come from a set of notes from an A&E department, but from a sickness benefit tribunal. However, the tribunal is centrally concerned with attendances at hospital A&E departments that result in the claimant being admitted to hospital. The benefits involved are National Health Insurance sickness benefits, but the question of eligibility for benefit straddles the admissions to hospital (which are conceived of as using “public money”), and the question of sickness benefit.

Finally, we turn to the man named at the start of this article, one William Spencer Davies. From what the files say, Davies served in the Royal Army Medical Corps between August 1940 and July 1942, before being invalided out for “psychopathic personality”.^13^ One of the Ministry of Health Officers preparing the case against him in the late 1950s accesses his War Record and claims that during this time “he was noted as a possible malingerer, a hypochondriac and a liar. … [H]e did not serve abroad and was never in action … [he complained of] imaginary abdominal pain and fake vomiting of blood … he was under police surveillance at one stage for posing as a Commando hero.” After this rather inauspicious start, Davies was then employed as a Lorry Driver at some point after the Second World War, living in various places including Halifax, Liverpool and Vauxhall. He was imprisoned for a short time in the 1950s for (probable) non-payment of child maintenance (ibid).

Davies has gained a foothold in the historical record because of his prodigious number of claims for sickness benefit, for time spent undergoing tests for presumed renal colic. At some point the local insurance officers became suspicious and began to deny his claims, decisions which he then appealed. One such attempt was successful, and he continued to claim benefits for a further year or so. When his claims were again refused, he appealed again, and this time the decision to withhold benefit was upheld.

These decisions are included in the Insurance Commissioner’s Reported Decisions (precedents guiding current decisions on welfare), and there are two large files of correspondence relating to these decisions at the UK National Archives in Kew. This case is important because it is a practical example of how psychopathy *functions* as a label, in contrast to the definitional debates in Parliament, in medical journals, and in the evidence to a Royal Commission. It shows an intimate connection between ideas of psychopathy, diagnoses of Munchausen syndrome, and the boundaries of the welfare state, or social welfare. As mentioned earlier, one of the things that gives the psychopath concept utility (and thus a functional sense of coherence) is how it works *in practice* to police social provision. Munchausen syndrome is used here to guard against the “exploitation” of welfare resources.

Davies’ case, and these two decisions on his eligibility for welfare, continue to be used in DWP guidance as precedents for refusing social welfare payments for a certain kind of behavior that is understood in a particular manner. The principle is set out in a “Decision Makers Guide” for DWP staff as follows: “A hospital in-patient can be treated as having limited capability for work even if admitted only for investigation of symptoms unless the investigation reveals that admission was due to another factor such as a personality disorder” (DWP 2022, para 42074). In other words, a potential claimant is normally entitled to relevant benefits (treated as having limited capability for work) when admitted to hospital for tests (i.e. before it has been established by a medical professional that they are definitively “ill”). Crucially, *even if no illness is found*, whilst an inpatient, one is entitled to the benefits. This principle applies *unless* you are in hospital undergoing tests as a result of a personality disorder. There is a reference at the end of the above-quoted paragraph to “Reported Decisions”: R(S) 1/58 and R(S) 6/59. Reported Decisions in general are considered to be “of major importance, because they established or illustrated important general principles” (National Archives, 2009). They are printed and made publicly available in order to serve as guidance for future decisions. The two decisions referenced here are the two Davies decisions, functioning as a precedent in the sickness benefit system.

In an article published in 1966, psychiatrist J.C. Barker mentions a Munchausen case who is a long-distance lorry-driver, along with references to the two “Reported Decisions” cited by the DWP. Barker expands upon this connection between Munchausen and welfare systems, but he dissents from the view that there is an easy link between socialized medicine and Munchausen. He argues that because “examples of this condition have been reported from all over the world,” this suggests that “its incidence is unrelated to free hospitalization and the National Health Service” (Barker 1966, 66). From the point of view being considered here: Munchausen syndrome in particular and psychopathy in general are clearly linked to social provision, but not in the easy reactionary move that supposes that as soon as benefits are provided, people will endlessly exploit them. Instead, the relationship revolves around how such resources are imagined and how they are policed. The precise connection between this particular case and welfare decisions is illuminating because it connects the *anxieties* around welfare seen in the interwar period, with the NHS-based presentations of Munchausen patients after the Second World War.

This case also shows how the ambiguity and uncertainty in psychopathy is necessary. Munchausen Syndrome, as a manifestation of psychopathic personality, is considered to be a disease only insofar as it allows a claimant to be understood as a kind of person disqualified from eligibility for benefits. It is just enough of a disease to establish with credibility that the man’s symptoms are knowingly false, but not enough for him to count as having a legitimate illness under the National Insurance regulations. Instead, Munchausen syndrome is established (after considerable work by civil servants) to be “a defect of character”.^14^ (This assessment of “character” does the same kind of work as “personality disorder” today.) The ambiguity helps to police the award of funds, by designating a man to be faking illness in a manner that does not make him securely ill.

To reiterate the technicalities: the principle tested here is whether a patient is entitled to sickness benefit when admitted to hospital for tests. As stated above, the normal inference drawn is that one *is* entitled to claim, even if the tests end up showing nothing wrong. Thus, in this particular circumstance a person is considered both “incapable of work” and “entitled to sickness benefit” for the time he or she is admitted to hospital for tests. This holds *even if* the tests subsequently show that the patient is well. However, in Davies’ case, a local Insurance Officer became suspicious when Davies was hospitalized for tests twenty-three times between April and September 1955, and “on only two occasions was he in the same hospital”.^15^ The local officer denied benefits on the basis that the claimant had not “proved that he was incapable of work” simply by being in hospital for tests.^16^ This decision goes against “the normal inference from the evidence that the claimant was an in-patient in hospital, namely, that he was incapable of work during the periods in question”.^17^ The case was therefore referred to a local tribunal.

The local tribunal made sure to emphasize that they had examined this story very closely, but continued to draw the standard inference that when a person is in hospital, they are incapable of work, and should be entitled to sickness benefit. It “could not conceive that a man could go about in this way and spend both long and short periods in hospital over a long period and yet be regarded as capable of work.”^18^ The local tribunal overruled the Insurance Officer’s denial of benefit on this basis—it was literally inconceivable for the tribunal that a person could be getting constant hospital tests and not be in a position to receive benefit: they could not see any ulterior motive. What is crucial here is that the lack of a credible *motive*—something that could be supplied by a psychiatric diagnosis—is what turned the case in Davies’ favor. The case was sent by the Insurance Officer to one final appeal, this time to an Insurance Commissioner.

The Commissioner, Archibald Safford, examined the case in an oral hearing in May 1957. He was rather more suspicious, given that the case had been appealed twice. He noted that having been discharged as fit for work on one day, the claimant was then “admitted to another hospital in another (and sometimes a distant) town either that same day or very shortly afterwards,” adding that “this pattern of behaviour appeared incompatible with the reasonable desire of a man in chronic ill-health to seek professional advice and systematic treatment”.^19^ However, the Commissioner also argued that “a man cannot be expected to work, if there are reasonable grounds for belief that he is suffering from a disease, while the matter is under investigation in hospital.” This point, however, turns on the question of what constitute “reasonable grounds,” and here the Commissioner conceded that “there may be cases where the evidence looked at as a whole justifies the inference that … the claimant in going to hospital has an ulterior object in view unconnected with health.”

The Commissioner’s response was somewhat complicated by the fact that he was not allowed to see reports from hospitals. This is because medical advice has been given that “it would be detrimental to the claimant if their contents were disclosed to him”.^20^ Because the claimant was not allowed to see the hospital reports (because of the impact upon his health), the Insurance Commissioner was reluctant to look at them, as this was only allowed in exceptional circumstances. He argued that “with [his] hands tied by being unable to look and see what is in the hospital reports” he must follow the normal inference that the claimant was incapable for work.

Safford concluded that “the facts undoubtedly raise the suspicion that the claimant’s protestations of pain are unfounded,” but that “on the other hand, some of the investigations in which he involved himself by reason of these allegations [of renal colic] can scarcely have been pleasant.”^21^ Here again, these is a sense that a person might be faking, but this is countered by the seeming unlikelihood of a patient undergoing painful procedures unnecessarily and by choice. Whilst there is suspicion of dishonesty, it is overridden by incomprehension that there could be any other motive (similar to Asher’s initial bewilderment), and an unwillingness to look at hospital reports. Thus, declared the Commissioner, because “I am not allowed to know what the hospital investigations showed, I must dismiss the insurance officer’s appeal.”^22^ Davies was allowed to keep claiming sickness benefit for time spent in hospital undergoing tests. His claims were allowed because the medical evidence was not admitted for consideration, but also because *there was no credible motive* to make this behavior make sense, other than a genuinely experienced medical condition on Davies’ part.

Before long, however, Davies’ case was back before Commissioner Safford, this time from a Leeds area tribunal. The lack of access to medical records had been overcome, because Davies had given his consent to their release. The lack of comprehension regarding the motives was solved by the testimony at the hearing of Dr. Frederick Collins, the Deputy Chief Medical Officer at the Ministry of Pensions and National Insurance.

The role of Medical Officers is central more generally to the National Insurance. As Arthur Massey (Collins’ direct superior, the Chief Medical Officer) wrote in Collins’ obituary in 1973, when Collins was appointed in 1947 to the Ministry of Pensions and National Insurance, he “helped me in forming and developing the new medical department which was established to deal with the wide medical aspects” that attended the new National Insurance regime at the time (Massey 1973, 742). Medical knowledge and medical assessment are central to insurance and compensation schemes going back to the late nineteenth century and Workmen’s Compensation Acts (Figlio 1982).

Collins’ testimony resolves Davies’ case by bringing in the diagnosis of a particular kind of psychopathy (Munchausen Syndrome), backed by a number of assiduously prepared appendices to the case material. One contains material transcribed from *The Lancet* and *British Medical Journal*: two full articles, fourteen letters and one report of a spoken address, all concerning Munchausen syndrome.^23^ Another appendix contains a long list of the various hospitals Davies had attended since the mid-1950s. Still another contains copies of the medical reports from the hospitals describing the man, as well as some more forthright letters between hospitals about him, voicing their suspicions that he is not really ill. In a note, Collins expresses some exasperation that the appendix with the medical journal transcripts (which are publicly available and thus not subject to any confidentiality ruling) was not properly considered at the time of the first appeal:

On 6^th^ May 1957 at considerable labour I reproduced correspondence which had appeared in “The Lancet” for a period of years on Munchausen’s Disease [sic]. I suggested that this should be put before the commissioner at the first hearing, but for some reason this was not done with the result that the Commissioner was not made aware of the existence of Munchausen’s disease, and the case was lost. The medical Assessor who sat with him did not seem to appreciate that the case was a typical Munchausen.^24^

Safford’s new decision, when hearing the case again in 1959, reports that the medical officer explained that “the claimant’s case was a typical case of Munchausen’s syndrome. This is a strange condition in the nature of malingering and the motive has never been clearly ascertained”.^25^ This response suggests that, in one sense, the Munchausen diagnosis has *not* supplied the motive. Rather, “Munchausen” functions as a kind of person, an intelligible kind of character in place of a formal motive.

## A behavior disorder, not a mental disease

This negotiation is seen clearly in a discussion between Collins and colleagues within the file notes. They realize that there is some work to do in order to make this particular category function in the welfare system. On 19 November 1958 it is suggested that “in the interests of being more intelligible to the layman … there be substituted ‘This is a behaviour disorder but there is no evidence of mental disease. The pains complained of are not imaginary in the sense that the claimant believes he has such pains. In fact, he knows he does not feel what he complains of’.”^26^ Five days later, on 24 November, further clarification is proposed: “‘The patient’s complaints are normally untrue and are known by him to be untrue…’ The one point I wish to make quite clear is that these are not cases in which the patients are deluding themselves” (ibid). There is clear management of ambiguity here: this is an illness, but not *that* kind of illness. This idea of “a behavior disorder, but not a mental disease” is a form of words that we will revisit in the conclusion, as very similar formulations keep cropping up around personality disorders and antisocial behavior orders—again the interaction of the legal system and the psychiatric category causing problems.

The sense of the novelty of this condition is clear, as Collins writes to his colleagues: “On the last occasion neither he [Commissioner Safford] nor his medical assessor seemed to know about Munchausen—he could not be expected to know of it unless someone told him!”(ibid.) Collins’ actions have the effect of folding Asher’s account of Munchausen syndrome (itself a condition framed in order to “protect hospitals” at A&E) into another part of the welfare state—as a reported decision, a functioning precedent—in a way that still operates today. It is not simply that Asher’s Munchausen article is sitting in an appendix to a welfare decision, or even that the information was clearly used in the hearing: the recorded decision adopts the structure and much of the wording of Asher’s article when discussing Munchausen. Munchausen emerges from a concern with resources and welfare, and serves to police it, to plug the gap through which this claimant had emerged.

The idea that the presence of this new syndrome might entitle the claimant to sickness benefit is definitively disallowed in the Recorded Decision, in the precise manner foreshadowed in the minutes of the Ministry of Pensions’ discussion:

In the medical officer’s opinion, the condition known as Munchausen’s syndrome was a defect of character, rather than a mental disorder. Persons with Munchausen’s syndrome knew that there was nothing wrong with them; in that respect they were different from the true psychotic, who was not really aware of his condition.^27^

The Insurance Commissioner and the Deputy Chief Medical Officer utilized the ambiguity in Munchausen and psychopathy. Indeed, one of Collins’ notes for the file is entitled: “Munchausen’s Disease or Syndrome (Psychopathic Malingering).”^28^ It is presented as a recognized, credible disorder with a clinical basis, but also bound up with malingering (fakery) and psychopathy. Its significance is that the normal inference of incapacity should not be drawn from the mere fact of being a hospital inpatient. What is crucial (and ambiguous) here is that it is also not *really* a disorder (mental or otherwise) but a defect of character. The multivalence and paradoxical nature of Munchausen make it a perfect fit for this purpose. This is because of the intimate connections often drawn in the twentieth century between the distribution of public funds and the image of the chronic scrounger, representing a shiftless class of people employing deception to maintain their status as permanent welfare recipients. As noted in the Jackson-Jones discussion, this category operates outside medical diagnostics, and it should be emphasized that Davies has broken no laws.

This latter point—that this kind of behavior is not illegal—is made clear in one doctor’s observation of a different Munchausen patient in 1955, who noted that "all the rumpus and cost to the Health Service were caused by the many doctors who ordered expensive investigations and treatment, and not by the patient, who merely, and quite lawfully, presented to his medical advisers with a tall story and a blood-stained face” (Clyne 1959, 1207). Thus the behavior is *lawful*, but still considered exploitative of hospital resources. William Spencer Davies broke no law by claiming benefits for time spent in hospital undergoing tests. However, the various local and regional insurance officers felt the need to prevent him from claiming funds.^29^ One of the letters between hospitals copied into Davies’ case file laments: “It is unfortunate that he is now free to go about the country gaining admission in various hospitals and it would be nice to be able to prevent this if only we knew how.”^30^ The desire to stop this patient from lawfully being admitted to hospital, and from claiming funds for his incapacity whilst being investigated, requires something productively ambiguous—a disease that is not quite a disease, an assessment of a “defect of character” that has the stamp of medical authority.

Thus Collins, the Deputy Chief Medical Officer, was backed by the medical assessor in claiming that this is a case of Munchausen, and that this should be regarded as a character defect. Further, the medical assessor “was not prepared to regard the condition as either a disease or a mental disablement.” Commissioner Safford concluded by saying that if “despite the absence of organic disease, it was reasonable to infer that the symptoms of which he complained were *real to him* with the result that he attended hospital in the genuine belief that benefit might be derived from doing so,” then he could hold to the view that the tests rendered the claimant incapable of work. On the basis of the diagnosis of Munchausen syndrome, this was denied. Thus, the fact that “in some cases he was rendered incapable of work by investigations which he caused to be carried out at the hospitals” was considered irrelevant. The claimant’s appeal was dismissed.^31^

As part of the proceedings described above, Asher’s category was literally transcribed at considerable effort from the *Lancet* and pulled right into the heart of the welfare system, where it remains to this day. It sits as part of the guidelines for those policing benefit claims, which determine that a “personality disorder” is not considered to be a “real” disorder or illness for these purposes.

## Conclusion

We have seen how uncertainty and ambiguity are used to deny a patient’s claim on welfare resources, in a pair of cases that remain part of the welfare system in Britain. This uncertainty is not a neutral tool, but is used by institutions against individuals, rather than the other way around. It is those officials with power to dispense or withhold funds, care or other resources, or to arrest and even imprison, who have the opportunity to use this uncertainty and ambiguity. There are some broader issues to be broached here around the intersection of psychiatric expertise and the policing of welfare resources. These problems have not gone away, even though William Spencer Davies is most likely long dead. Serious controversy over the use of the category “personality disorder” remains.

Over twenty years ago the National Health Service report “*Personality Disorder: No longer a diagnosis of exclusion*” (2003) addressed this idea that a diagnosis of personality disorder was sometimes used to exclude people from care, although this was assumed to be on the grounds that they did not have appropriate expertise to offer treatment, rather than any explicit statement that the patient was not deserving of it. Service user contributions to that report show that “no mental disorder carries a greater stigma than the diagnosis ‘Personality Disorder’” and people with this label “have been described as ‘the patients psychiatrists dislike,’ and many reported being called time-wasters, difficult, manipulative, bed-wasters or attention-seeking” (National Institute for Mental Health England 2003, 20).

Despite a general tone that seeks to include and legitimize personality disorders, the report mentions the demands on services in a way that would be unthinkable for say, a cancer patient: “What is clear is that people with personality disorders make heavy demands on local services. … They tend to have relatively frequent, often escalating, contact across a spectrum of services” (ibid., 12) Interestingly, service users diagnosed with personality disorders also report being told “you’re not mentally ill,” once again showing the reluctance to definitively code personality disorders as mental illnesses, even amongst healthcare professionals (ibid., 20). These reports echo statements such as “This is a behaviour disorder but there is no evidence of mental disease” found in the Ministry of Pensions files from the 1950s.

Around the same time, there was more controversy concerning Anti-Social Behavior Orders (ASBOs), which were part of New Labour’s flagship criminal justice legislation in the Crime and Disorder Act of 1998. The recipient of such an Order “must be at least 10 years of age, he must have acted in ‘an anti-social manner, that is to say, in a manner that caused or was likely to cause harassment, alarm or distress to one or more persons not of the same household as himself’” (Macdonald 2006, 184). Here again was a problem of antisociality which (seemingly) required a recasting of the law to meet it, recalling the discussion between Meredith Jackson and Maxwell Jones forty years before. Further, breach of the terms of any ASBO “without reasonable excuse” became a breach of the criminal law “punishable by up to five years’ imprisonment and/or a fine” (ibid.).

Stuart Macdonald’s careful analysis of the operation of these ASBOs brought him to the case of Kim Sutton, who was subject to one preventing her from “from jumping into rivers, canals or onto railway lines after she had attempted suicide on four occasions” (Macdonald, 2006, 199). Macdonald raised concerns at what he saw as a recriminalization of suicidal behavior (decriminalized in 1961 in England and Wales). Sutton appealed this order in 2005, on grounds that are incredibly relevant to the ambiguity we have been discussing. Her legal team argued that “her personality disorder meant that she needed help and that legal sanctions could in fact be counter-productive” (ibid.). This argument for care was rejected and Sutton lost the appeal. The BBC reported that “the 23-year-old is not mentally ill, but does have a personality disorder” (BBC, 2005). The echoes of “this is a behaviour disorder but there is no evidence of mental disease” are striking. Ambiguity is managed, care is withheld.

The example of “Serenity Integrated Mentoring” (SIM) shows how these problems persist right up to the present day, and how this ambiguity within the concept of “personality disorders” continues to enact the exclusion of patients from care in a different configuration. (Much of what follows relies heavily upon the research done by a group called the StopSIM Coalition, who have raised serious concerns about SIM.^32^)

A recent editorial in the British Medical Journal approaches the problem from the perspective of suicidality and suicidal crisis (and includes references to the Sutton case). In short:

SIM aims to reduce demand on emergency services by allocating a police officer “mentor” taking the role of a mental health key worker for regular meetings. In the event of further emergency service call-outs, the SIM operational delivery guide includes a section titled “Use of criminal and behavioural sanctions” and guidance on framing distress during psychiatric emergencies as a criminal offence, describing such distress as, “behaviours that whilst not substantive offences in their own right, would be considered disorderly or antisocial for the purposes of any criminal or civil court order.” (Thompson et al. 2022)

Thus care is withdrawn, resources are “protected,” and criminal sanctions are threatened, once the case is processed through ideas recasting antisocial behavior as a criminal offence. Patients subject to SIM are clearly thought overwhelmingly to have Borderline Personality Disorder (BPD), because the SIM Operational Delivery Guide states more than once that “SIM mentoring is based upon guidelines from NICE for working with Borderline Personality Disorder” (SIM Operational Delivery Guide, n.d., 14, 29). In this instance too, we have personality disorder, demand upon healthcare resources, and the criminal justice system, using updated definitions of ASBOs (Thompson et al. 2021, 3). Antisociality, a recasting (expansion) of the law to encompass people diagnosed as mentally ill, but with *that category again* (or its successor, to be precise) using ambiguity to blur these lines in the project of denying welfare and healthcare resources.

This blurring or uncertainty is central to the operation of these teams, who use the ambiguity of the term “personality disorders” to claim that a person is ill, so that they come under their purview, but then modify the *kind* of illness to justify the withdrawal of support (sometimes, as in the case of SIM, this withdrawal itself is coded as “therapeutic.” This is precisely the multivalence of Munchausen (and personality disorders more generally): they are medical conditions for the purpose of credibly disqualifying a person from claiming a certain kind of benefit, but they do not count as medical conditions for any other purpose (such as the actual provision of healthcare, rather than its withdrawal). Viewed from this perspective, “inconsistency” looks much more like *necessary uncertainty*.

It is of course very likely that personality disorders are highly contested because psychiatrists are split and uncertain about them, and they have such huge implications. Part of the uncertainty and contestation doubtless arises because the stakes are so high, and the evidence so scattered and inconsistent. But the uncertainty goes deeper than that, and is more *useful* than that. The diagnosis must *include* in order to understand, to categorize and to label, and then have the capacity to *exclude* when it comes to healthcare or welfare resources. For one final reminder: the civil servants trying to convince the Welfare Commissioner formulated it thus: “This is a behaviour disorder but there is no evidence of mental disease.”

The ambiguity, or “ontological anarchy,” of the concept of psychopathy has been illustrated here via the dual examples of the of Percy Commission evidence and recently-released National Insurance Sickness Benefit files relating to William Spencer Davies. These materials show how the abstract definitional discussions of psychopathy were developed through the functional minutiae of one particular claimant’s multiple appeals. In them, the uncertainty of psychopathy is not a problem to be solved, but a powerful tool of policing to be judged and contextualized. This concept’s disciplining of a kind of individual—the “vagrant scrounger”, or the “attention-seeking suicide attempter”—gives it a practical coherence. This includes, but is not limited to, denying welfare payments. Thus, a paradox of medical authority is created, which simultaneously both confirms *and* denies the validity and legitimacy of Munchausen Syndrome.

If we wish to understand diagnoses of psychopathy in the 1950s, or personality disorder today, as more than simply incoherent or illegitimate (although they may well be both), we might start by looking at what these conditions *do* rather than what it *are*. If we worry (and we should worry) about the legitimacy of withdrawing treatment from those diagnosed with “personality disorders” because they are “high intensity users” of health services, it is worth digging into the kinds of categories that animate and legitimize practices at these fragile system edges where welfare, criminal justice and psychiatry bleed into each other. The exclusionary power of this diagnosis is characterized by its ambiguity and multivalence. It is a protean authority where gaps are filled in and institutional or regulatory limitations are overcome. It is power founded on necessary uncertainty and productive ambiguity.

## Footnotes

1 In the early 2020s, an initiative called Serenity Integrated Mentoring (SIM) attracted much criticism for its approach, which brings in the police and threats of criminal sanction in order to change the behavior of people considered to have personality disorders. SIM coded these behaviors as “attention seeking” and actively sought to withdraw resources (such as inpatient admission) from those said to exhibit them. This is explored further at the end of this article.

2 It was not until the late 1960s that these patients became understood (by sociologists initially) as wanting to inhabit “the sick role” (see Pilowsky 1969).

3 Leo Lamphere is first reported by Chapman (1955); in contrast, one of the last articles on Samuel McIlroy is by Pallis and Bamji (1979).

4 I acknowledge that this link has been made (with material effects) rather than validating such a link.

5 Mather (2019) cites Brashers (2001) and Matthias (2010) as examples of these positive attitudes to uncertainty.

6 See Pickersgill (2014, 153–154) on the Butler Report (1975) which recommended the abandonment of the term “psychopathy” and its replacement by “personality disorder”.

7 Pickersgill does not entirely rule this out: “a stable referent for terms such as ‘psychopathy’ or ‘antisocial personality disorder’ has so far escaped the sophisticated attempts of psychiatrists to constitute it” (Pickersgill, 2014, 146).

8 HC Deb 08 July 1957 vol 573, col 38.

9 HC Deb 08 July 1957 vol 573 col 70.

10 Mental Health Act 1959, ch. 72 s 4(4).

11 This is presumably Henderson and Gillespie’s multi-edition *Textbook of Psychiatry*. Shapland notes that this analogy opens a 1944 article on “Psychopathic Personalities” by Curran and Mallinson. She also notes that this analogy “is cited approvingly by a number of other psychiatrists, suggesting it was considered a good analogy for the case of psychopathic personality” (Shapland 2019, 7).

12 See, for instance, Shane 1958, 533; Ekdawi & Lear 1961, 229.

13 TNA PIN 62/ 1485. Letter from Frederick M Collins to Ronald Hyam (25/09/1958)

14 CT 5 Sickness Benefit Decision R(S) 6/59.

15 CT 5 Sickness Benefit Decision R(S) 1/58.

16 In this, previous decisions of the Commissioner were drawn upon, specifically Decisions R(S) 1/53 and R(I) 13/55, “in which it was pointed out that the question whether a person was incapable of work was a question of fact and that a doctor’s certificate was not conclusive evidence of incapacity” (CT 5 Sickness Benefit Decision R(S) 1/58).

17 CT 5 Sickness Benefit Decision R(S) 1/58.

18 CT 5 Sickness Benefit Decision R(S) 1/58.

19 CT 5 Sickness Benefit Decision R(S) 1/58.

20 CT 5 Sickness Benefit Decision R(S) 1/58.

21 CT 5 Sickness Benefit Decision R(S) 1/58.

22 CT 5 Sickness Benefit Decision R(S) 1/58.

23 The “Address” (given by Asher) is entitled “Illness as A Hobby”, but all the others explicitly mention Munchausen Syndrome.

24 PIN 62/1485, 1957–1959 Frederick Collins, Note for File, 08.08.1958.

25 CT 5 Sickness Benefit Decision R(S) 6/59.

26 PIN 62/1485, 1957–1959 Mr. MacCabe, Note for file, 19.11.1958.

27 CT 5 Sickness Benefit Decision R(S) 6/59, 1959.

28 PIN 62/1485, 1957–1959 Frederick Collins Note for File 08.08.1958.

29 There are other recorded attempts to criminalize Munchausen patients by claiming that they defraud hospitals of food. One man was imprisoned for six months for this (anon 1957, 696).

30 PIN 62/1485, 1957–1959 Letter from Leicester Infirmary to Royal Halifax Infirmary, 08.04.1957. Transcription of Documents Held by Insurance Officer.

31 CT 5 Sickness Benefit Decision R(S) 6/59, 1959.

32 For more information, see: “Stop Sim: Mental Illness is Not a Crime” at https://stopsim.co.uk/ (last accessed 16.09.2022).
